# Comparing the seoi-nage skill of elite and non-elite judo athletes

**DOI:** 10.1038/s41598-023-49188-w

**Published:** 2023-12-07

**Authors:** Sang-Hyup Choi, Yong-Gwan Song

**Affiliations:** 1 Department of Physical Education, Korea University, Seoul, 02841 Republic of Korea; 2 Division of Smart Healthcare, Pukyong National University, 45, Yongso-ro, Nam-gu, Busan, 48513 Republic of Korea; 3 Department of Marine Design Convergence Engineering, Pukyong National University, Busan, 48513 Republic of Korea

**Keywords:** Human behaviour, Motor control

## Abstract

Seoi-nage performance requires a high level of skill and proficiency. The aim of this study was to compare the motor planning, regulation, and control skills of elite versus non-elite seoi-nage judo athletes. Twenty subjects (10 elites and 10 non-elite) performed the three-phase seoi-nage skills of unbalancing, positioning, and throwing while an optical motion capture 3D camera monitored their shoulder, pelvis, hip, and knee joint movements to calculate their force magnitude and direction. Elite athletes performed better than non-elite athletes in terms of the shoulder (247.4° vs. 208.3° in Event 4) and pelvic (235.4° vs. 194.4° in Event 4) rotation, tilt angle (15.13° vs. − 0.74° in Event 4) characteristics, as well as hip (136.1° vs. 125.0° in Event 4) and knee joint (124.0° vs. 120.8° in Event 3) flexion–extension angle. Compared to non-elite athletes, elite athletes also showed more controlled force and movement in all bodily areas. These results can help to guide the development of seoi-nage skills as judo athletes advance from the non-elite to the elite level.

## Introduction

The seoi-nage is one of the most important skills in judo. Seoi-nage skill is a movement skill, in which a judo athlete particularly uses to pull his or her opponent in different directions before throwing over one's back^1–6^. Seoi-nage labeled for efficient movement of upper and lower limbs is a difficult throwing technique since this complex skill form makes use of the degrees of freedom in the majority of the joints in the body^7,8^. The attacker's unbalance and the speed of the torso's rotation are both related to a high level of seoi-nage^5–7^.

Athletes should pull the opponent firmly, shake their center of gravity, and quickly swivel their torso to lift them in order to execute the seoi-nage skill properly. The throwing movement through seoi-nage is quicker than that used by judo athletes according to expertise. Previous research indicated that elite athletes (i.e., experts) exhibited delayed response initiation and body and postural responses following external perturbations with compared to non-elite athletes (i.e., amateur^1,5,6^. Moreover, elite athletes tilt their bodies more, which allows for quick torso rotation. And enough forward pull facilitates quicker and more precise placement^1^.

When the athlete pulls an opponent down with one’s hands to create his reaction, he or she can directly trip up with opponent’s legs ffective throwing^1–5^. The positioning and throwing procedure is carried out in the unbalancing situation (i.e., with the opponent’s center of gravity up and forward), hence the unbalancing direction is crucial. The increasing amount of frontal momentum of the opponent causes the performance speed of seoi-nage to look faster during accurate unbalancing and positioning^9^. Unbalancing, positioning, and throwing in performing seoi-nagi skills, more quickly and successfully, they should all be continuously and organically connected and appear as one movement.

In the elite athletes, the angular velocity and center of gravity in the vertical direction were important^1^. Elite athletes during seoi-nage, the center of gravity that appeared during each phase (unbalancing, positioning, and throwing) move forward and upward during the unbalanced positioning phase and downward during the throwing phase^9–11^. In addition, elite athletes are likely to use more trunk joint angular velocity during the rotating phase than do non-elite athletes, while femoral joint angular velocities in both elite and non-elite athlates were comparable^12^. Such findings provided important insight in the literature in which the phases were split and kinetic movement analyses were conducted with usage of 3-D image^10–12^; However, the kinetic variables, called kinetic movement, have not been examined for the force magnitude in the peak performance of the seoi-nage.

The strength of the tilting force in seoi-nage is crucial because jigging and hanging are only possible when the opponent’s center of gravity is slanted up and in front of them. It's also significant how the pulling force changes in strength as the phases advanced. Attempt to identify features in the kinematics and magnitude of force is important for better understanding of seoi-nage skills. Yet, the mechanisam of execution of seoi-nage skill is unclear and its applicability is limited in the current literature. No studies that have looked at the factors together. Furthermore, judo-specific device is required to/is considered to measure the single-axis pulling force and the amplitude of the force in each phase of techniques in the execution of seoi-nage, as well as measuring kinematics.

Several studies to understand judo skills in soei-nage have been conducted, but little is known to explain the mechanism how athletes with different levels of skills and execution demands perform in the process of motor skill acquistion. It has been reported that the seoi-nage is crucially played in the adaptability of body/balance control when postural stability is perturbed. However, specific kinematic characteristics of seoi-nage particularly associated with skill level are still unknown.

The aim of the present study was to examine the kinematic characteristics of seoi-nage on body/balance control in response to skill level. In this study, the kinematic variables of seoi-nage were simultaneously examined in three dimensions while the force magnitude was measured by suspending a rubber tube for judo training in judo-specific equipment. Our study differs from earlier ones in particular by simultaneously analyzing kinematic features and measuring the force value of seoi-nage using specific equipment. Our hypothesis was that, in a period of seoi-nege, non-elite athletes would demonstrate bigger kinematic deficits than elite athletes do. Consequently, these results can probably be used as factual information to improve the efficiency of judo seoi-nage learning and to further serve as the foundation for precise training recommendations.

## Materials and methods

### Participants

Partcipants in this study were twenty Judo athletes. All athletes were ethnic Korean. On average, elite athletes were 20.9 years old (*SD* = 0.88; *range* = 20–22) and had 7.7 years (*SD* = 2.54) of athletic experiences. On average, non-elite athletes were 27.5 years old (*SD* = 2.68; *range* = 20–29) and had 4.7 years (*SD* = 2.50) of athletic experiences. As shown in Table 1, we assigned them into ether elite athlete group (EG; n = 10) or non-elite athlete group (NG; n = 10). All participants had no history of neurologic disease or musculoskeletal dysfunction and had no prior experience with the experimental task and were not aware of the specific purpose of the study. All participants were right-hand dominant, as determined by self-report prior to the experiment. In this study, the criteria for participants recruitment whether athletes were included are as follows: Elite athletes who had the experience of international competitiions as national representitives for Korea were included in elite athletes group, while non-elite athlete had no experience of international competitions at all. All participants were requested to understand the purpose of this study for voluntary participation. The t-test on the height and weight of athlete participants in both EG and NG showed no significant difference in the height (*t* = -0.40, *p* = 0.696) and body weight (*t* = 1.31, *p* = 0.206) between EG and NG. In addition, using the G*Power 3.1.9.7 program, the sample was calculated with an effect size of 0.30, a significance level of 0.05, and a power of 0.95 required to test the difference. As a result, a minimum of 19 research participants were required.

**Table 1:** Characteristics of participants (mean ± standard deviation, N = 20).

Group	Age (years)	Height (cm)	Weight (kg)	Career (years)
Elite-group (EG; n = 10)	20.9 (0.88)	173.6 (6.42)	80.8 (14.54)	7.7 (2.54)
Non-elite group (NG; n = 10)	27.5 (2.68)	174.7 (6.00)	73.4 (10.38)	4.7 (2.50)

The protocol was approved by the Institutional Review Board of Pukyong National University (1041386-2023-HR-39-01) and conformed to the Declaration of Helsinki. All aspects were conducted in accordance with the relevant guidelines and regulations of the institution. experimental Informed consent was obtained from each subject before participation in the experiment.

### Apparatus and task

**Figure 1:**
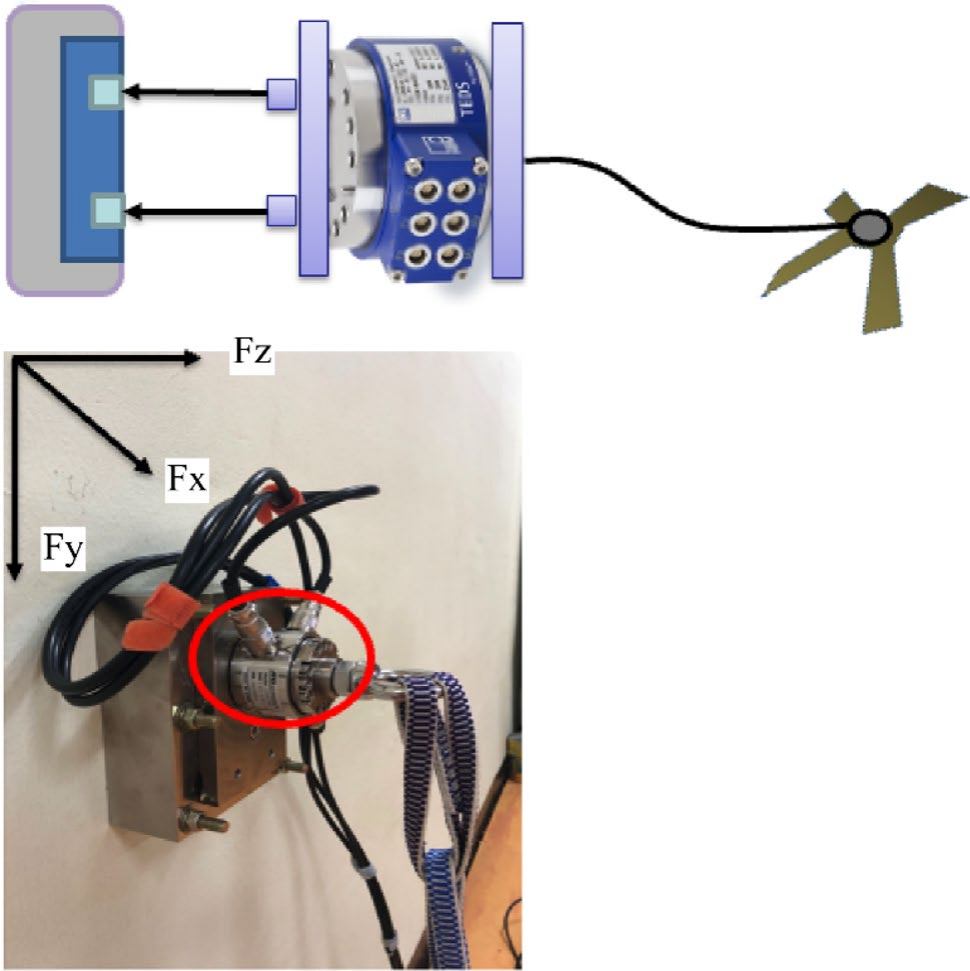
Vector analysis device. After fixing the judo vector equipment to the wall, the judo rubber tube is hung on the load cell hook to perform shoulder throw. The pulling force and direction are measured simultaneously.

A vector system was used to measure the magnitude and direction of the force. A three-axis load cell sensor was used to measure the magnitude and direction of the force on the Z-axis approximately 9800 N (in 1 ton), X and Y-axis approximately 4900 N (in 500 kg), and nonlinearity (1%). The maximum yield load was 150%. While the Z-axis measured the subject in the horizontal direction, the Y-axis and X-axis measured those in vertical and horizontal directions. We measured the magnitude of force appearing in the X, Y, and Z directions and the sum of the force vectors at a sampling frequency of 200 Hz (Fig. 1). The rubber tube connected to the judo throw analysis device was held with both hands to perform seoi-nage at maximum speed and force. The baseline was similar for the rubber tube and both arms. The equipment was located 80 cm from the ground, and the distance between the participant and the equipment was 150 cm.

We used an optical motion capture camera (Optitrack, Natural Point, Inc. USA) system to analyze the 3-D images recorded during seoi-nage. The subjects’ kinematic variables, i.e., shoulder and pelvis rotation angle (counterclockwise rotation + value, clockwise rotation − value), shoulder and pelvis tilt angle (posterior tilt + value, anterior tilt − value), and hip and knee joint angles (extension + value, flexion − value), were measured using 20 cameras. The motion capture camera's frame rate was 120 frames per second, and reflective markers were positioned on the head, shoulder, hip, knee, ankle, and posterior superior iliac spine to measure the performance. A marker was attached to the seventh cervical spine to distinguish the center of the body and on the lower scapula to differentiate the left and right sides. Before the task, a suit with a marker was attached for the 3-D image analysis. We attached the markers to each position and placed them at the baseline to hold the rubber tube connected to the judo throw equipment. Both groups participated in a series of seoi-nages, with two practice attempts. A baseline was set at a distance of 150 cm from the judo throw equipment to identify the performance position. Subsequently, on indicating a ready sign, the participants performed seoi-nage with maximum force and speed. The task involved performing seoi-nage five times, with a 30-s interval between each attempt. The kinematic data measured using the Optitrack motion capture program were digitized by signal cleaning through C motion’s 3-D 5 V professional, and the quantified data were normalized using MATLAB (The MathWorks Inc., R2015b, Version 8.6, Massachusetts, USA).

**Figure 2:**
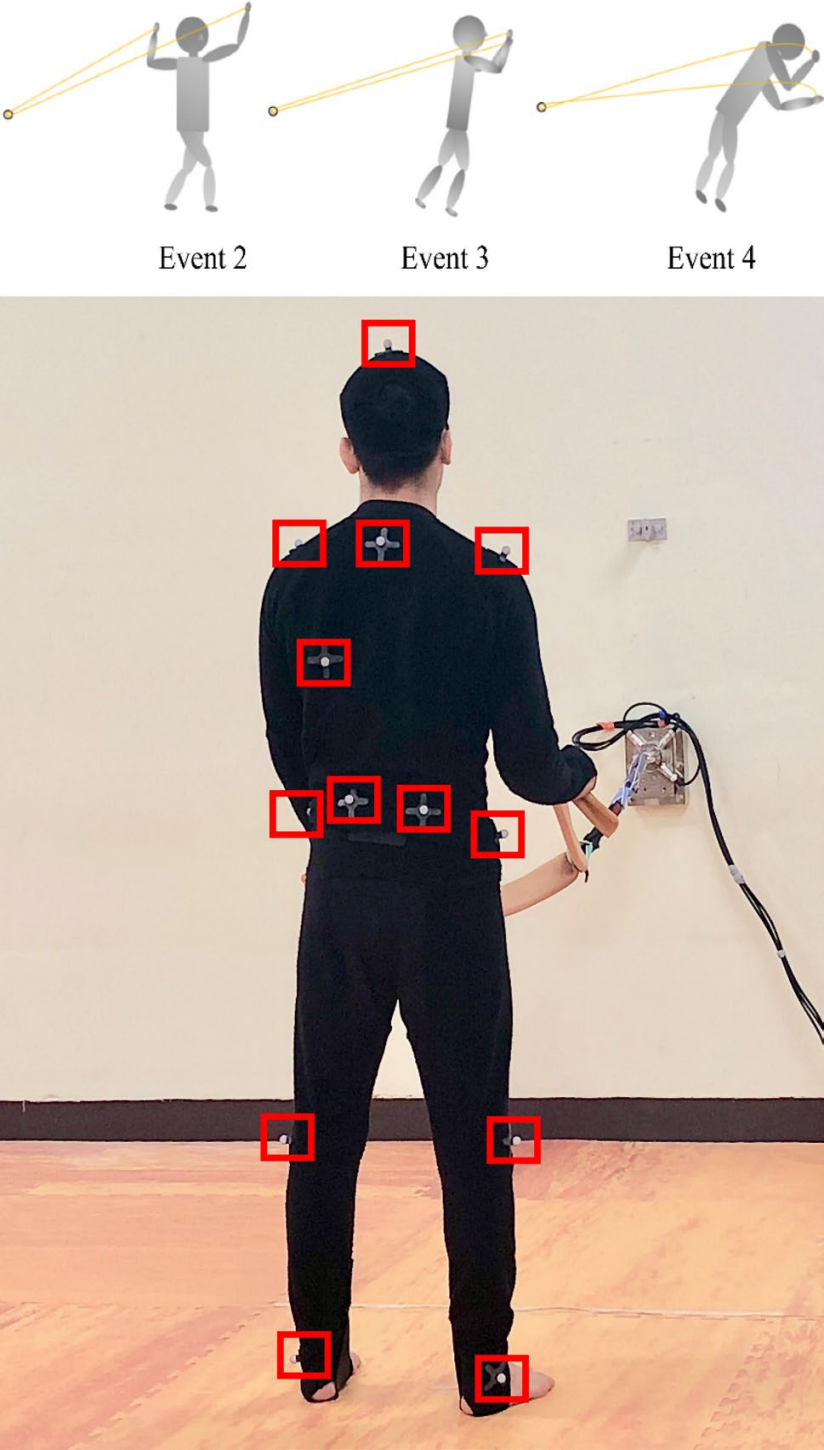
Model diagram for each event and marker position.

**Figure 3:**
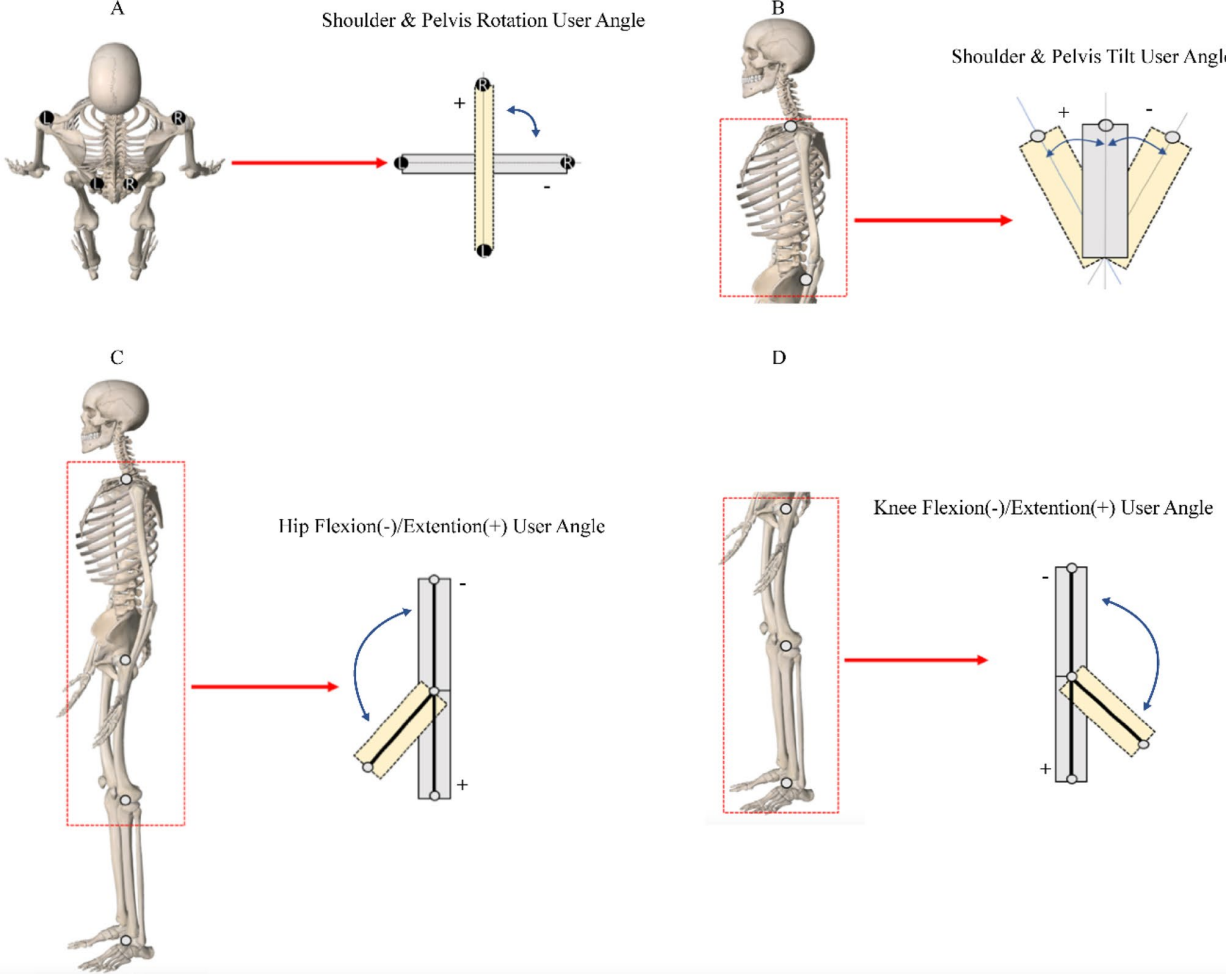
The kinematic variant diagram. (**A**) Horizontal angle of rotation of the shoulder and pelvis; (**B**) tilt angle of the shoulder and pelvis; (**C**) angle of flexion–extension of the hip joint; (**D**) angle of flexion–extension of the knee joint.

The kinematic characteristic data collected during seoi-nage were classified into four events (Fig. 2). First, the beginning of seoi-nage and first left foot contact time was set as Event 1, following which the left foot rotated and landed, defined as Event 2 (unbalancing phase). Event 3 was set as the point where the shoulder’s horizontal rotational angle reached 180° (positioning phase). Event 4 was set as the point when the horizontal rotational angle of the shoulder reached its maximum (throw phase). Event 1 was defined as the motion of stepping forward with the right foot before pulling the judo rubber tube from the stationary position and was excluded from the analysis owing to the absence of pulling motion. The analyzed kinematic variables were the shoulder and pelvis rotation angle (counterclockwise rotation + value, clockwise rotation − value), shoulder and pelvis tilt angle (posterior tilt + value, anterior tilt − value), and hip and knee joint angles (extension: + value, flexion − value). Figure 3A–D depicts the respective definitions of the horizontal rotation angles of the shoulder and pelvis, tilt angles, and hip joint and knee joint flexion angles. In addition, we measured only the horizontal rotation angle of the shoulder and pelvis, tilt (front and back), and the flexion angle of the left hip and knee joints, which is the most important factor in seoi-nage movement. A total of 13 markers were attached to avoid obscuring the 3-D view. Therefore, we limited the difficulty in collecting kinematic data and decided that the motion factor of seoi-nage was sufficient as the factor mentioned above.

### Statistical analyses

Statistical analysis was performed separately for kinematic variables of the seoi-nage and performance associated with event motion. Each event was subjected to a separate independent *t*-test for EG and NG. For all analyses, statistical significance was set at a level of 0.05. We performed all analyses using SAS (SAS 9.1.2. software; SAS Institute, Inc., Cary, NC, USA).

### Ethics approval and consent to participate

The research was approved by the Institutional Review Board of Pukyong National University. And informed consent was obtained from each subject before participation in the experiment.

## Results

### Kinematic variables in seoi-nage

**Figure 4:**
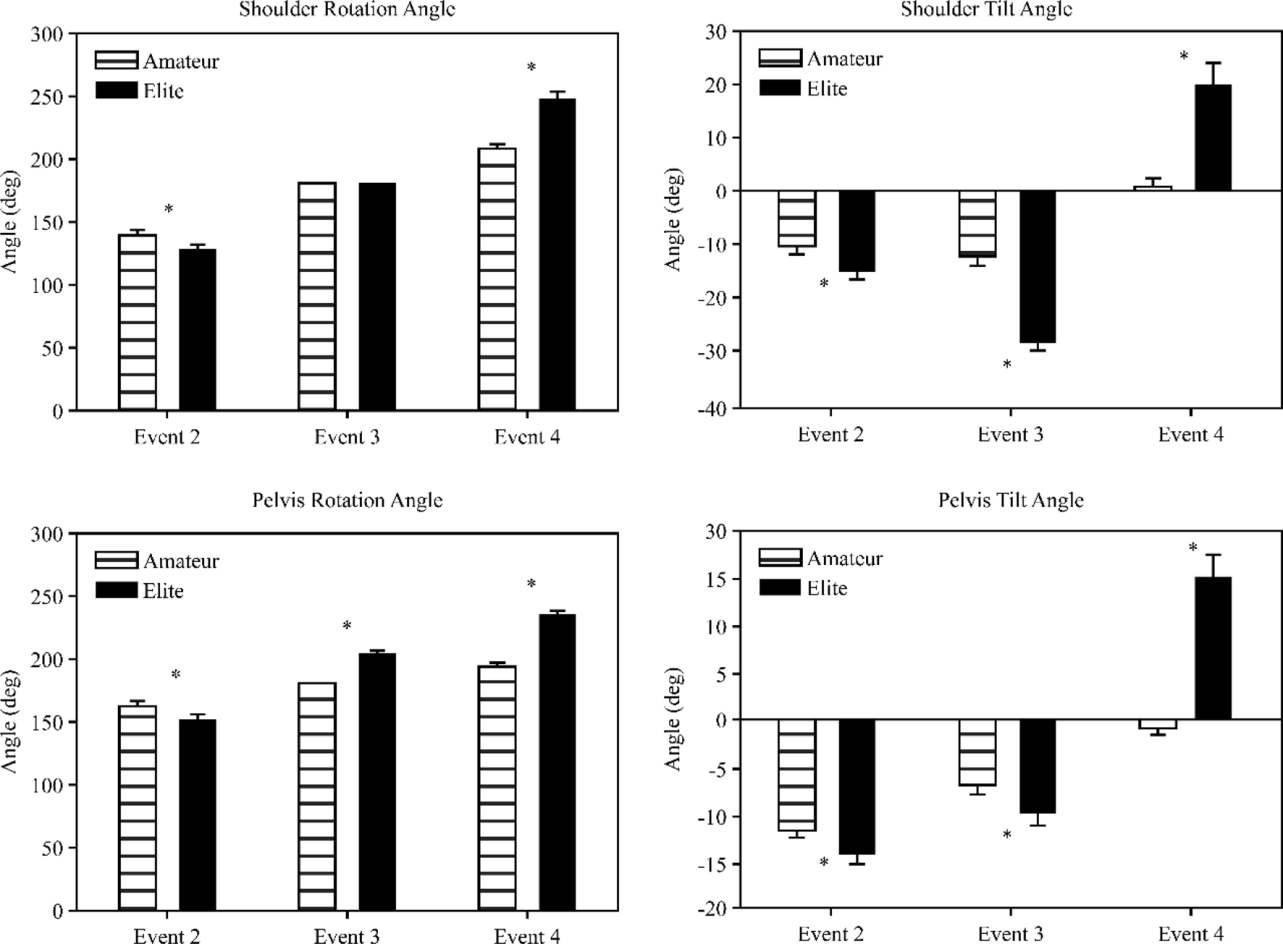
Kinematic variables in seoi-nage (shoulder and pelvis).

In Event 2, the horizontal rotation angle of the shoulder was 129.13° (SD = 20.37) and 138.9° (standard deviation [SD] = 26.12) in EG and NG, respectively (*t* = − 2.05, *p* < 0.05) (top left in Fig. 4). Following Event 3, the angle was 180°. Subsequently, the angle reached the maximum value in Event 4 and demonstrated a significant difference between EG and NG (247.4° [SD = 36.69] vs. 208.3° [SD = 18.54]) (*t* = 6.53, *p* < 0.01).

The anterior and posterior tilting angles of the shoulder (top right in Fig. 4) in Event 2 were − 15.13° (SD = 9.61) and − 10.42° (SD = 10.25) in EG and NG, respectively (*t* = − 2.31, *p* < 0.05). In Event 3, the angle was − 28.65° (SD = 6.40) and − 12.14° (SD = 12.66) in EG ang NG, respectively (*t* = − 8.04, *p* < 0.01). In Event 4, the tilt angle in EG and NG was significantly different (19.54° [SD = 29.23] vs. 0.62°]SD = 12.01]) (*t* = 4.13, *p* < 0.01).

In all events, EG exhibited a larger horizontal rotation angle of the pelvis than NG (bottom left in Fig. 4). In Event 2, the rotation angle in EG and NG was 163.9° (SD = 26.80) and 153.8° (SD = 22.25), respectively (*t* = 1.99, *p* < 0.05). In Event 3, there was a significant difference between EG and NG (205.7° [SD = 9.21] vs. 181.0° [SD = 10.47]) (*t* = 12.22, *p* < 0.01). Moreover, we observed a significant difference between EG and NG in Event 4 (235.4°[SD = 23.97] vs. 194.4° [SD = 15.71]) (*t* = 9.82, *p* < 0.01).

The anterior and posterior tilt angles of the pelvis (bottom right in Fig. 4) were significantly different between the groups (*t* = − 2.33, *p* < 0.05). In Event 3, the tilt angle in EG and NG was -9.91° (SD = 7.58) and − 6.72° (SD = 4.90), respectively (*t* = − 2.43, *p* < 0.05). In addition, the tilt angle in Event 4 was 15.13° (SD = 15.92) and − 0.74° (SD = 4.90) in EG and NG, respectively (*t* = 6.54, *p* < 0.01).

**Figure 5:**
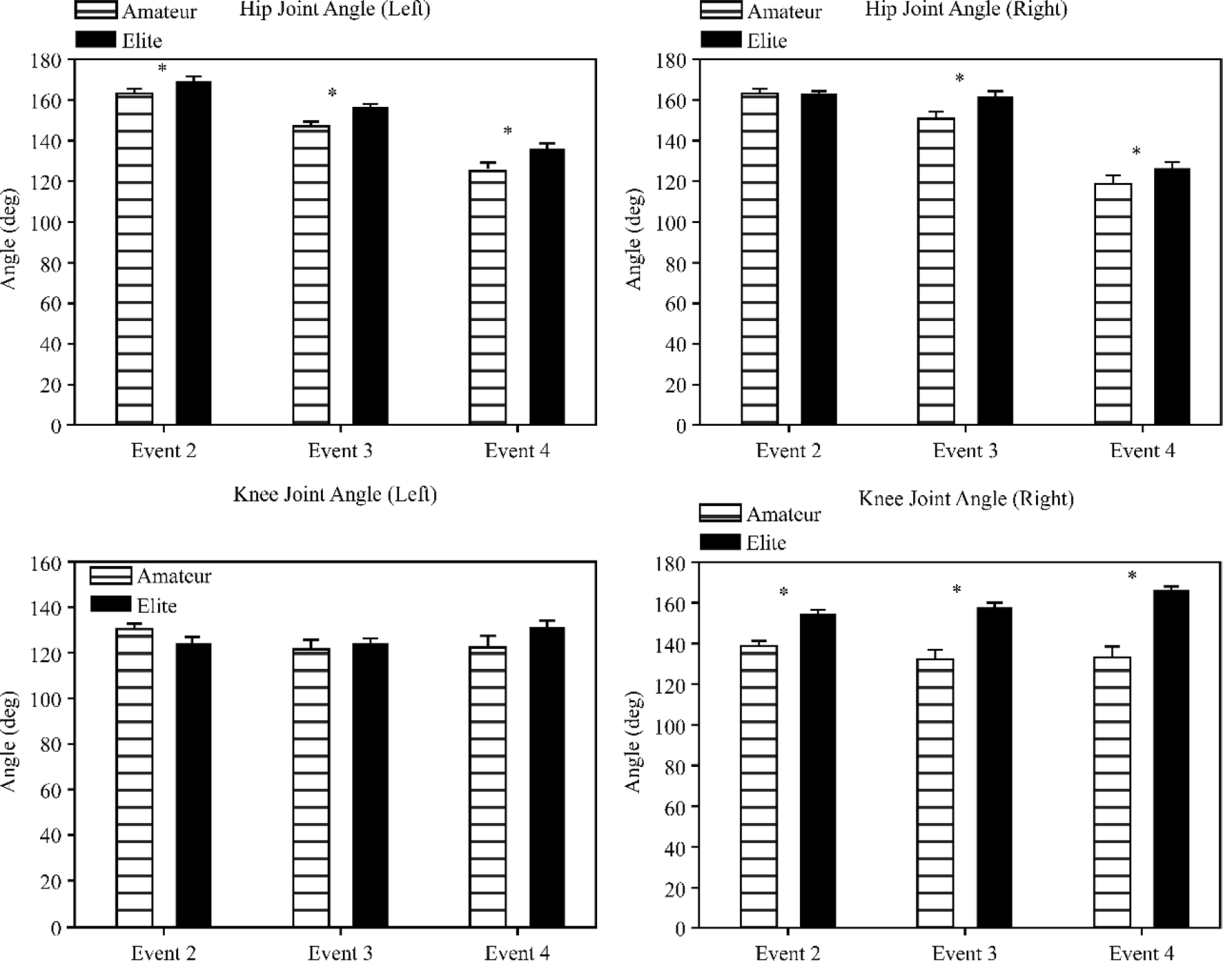
Kinematic variables in seoi-nage (hip and knee).

The angles of flexion (−) and extension (+) of the left hip joint were analyzed when performing seoi-nage (top left in Fig. 5). As a result, in Event 2, EG exhibited an angle of 169.8° (SD = 7.34) and NG demonstrated an angle of 163.3° (SD = 13.25) showed a significant difference between the two groups (*t* = 2.97, *p* < 0.01). In Event 3, EG showed an angle of 156.4° (SD = 10.30), and NG demonstrated an angle of 146.5° (SD = 23.10), and there was a significant difference between the two groups (*t* = 2.70, *p* < 0.01). In Event 4, similar to previous results, a significant difference was observed (*t* = 2.26, *p* < 0.05); EG showed an angle of 136.1° (SD = 14.98), and NG exhibited an angle of 125.0° (SD = 30.64).

There were no significant differences in the flexion (−) and extension (+) angles of the right hip joint (top right in Fig. 5) in Event 2 (*t* = − 1.40, *p* = 0.163). However, there were significant differences between the groups in Events 3 and 4. In Event 3, EG and NG demonstrated an angle of 163.0° (SD = 11.49) and 151.3° (SD = 26.52), respectively (*t* = 2.81, *p* < 0.01). In Event 4, EG and NG exhibited an angle of 127.2° (SD = 16.07) and 119.2° (SD = 29.95), respectively (*t* = 1.63, *p* = 0.105).

Differences in the flexion (−) and extension (+) angles of the left knee joint (bottom left in Fig. 5) were insignificant in Event 2 (EG, 124.7° [SD = 8.83] vs. NG, 129.8° [SD = 16.03]) (*t* = − 1.90, *p* = 0.06). In Event 3, the angle was 124.0° (SD = 10.38) and 120.8° (SD = 29.52) in EG and NG, respectively, without a significant difference between the groups (*t* = 0.72, *p* < 0.05). However, Event 4 did not demonstrate a significant difference (t = 1.81, p = 0.074) in the angles (EG, 131.3° [SD = 15.21] vs. NG, 121.7° [SD = 33.07]).

In Event 2, the flexion (−) and extension (+) angles of the right knee joint (bottom right in Fig. 5) were significantly different between the groups (*t* = 5.44, *p* < 0.01). In Event 3, EG and NG demonstrated angles of 157.1° (SD = 11.75) and 131.3° (SD = 30.97), respectively (*t* = 5.39, *p* < 0.01). In Event 4, the angle was 165.9° (SD = 9.78) and 132.6° (SD = 33.76) in EG and NG, respectively (*t* = 6.57, *p* < 0.01).

### Kinematic variables during maximum vector sum force expression while performing seoi-nage

**Figure 6:**
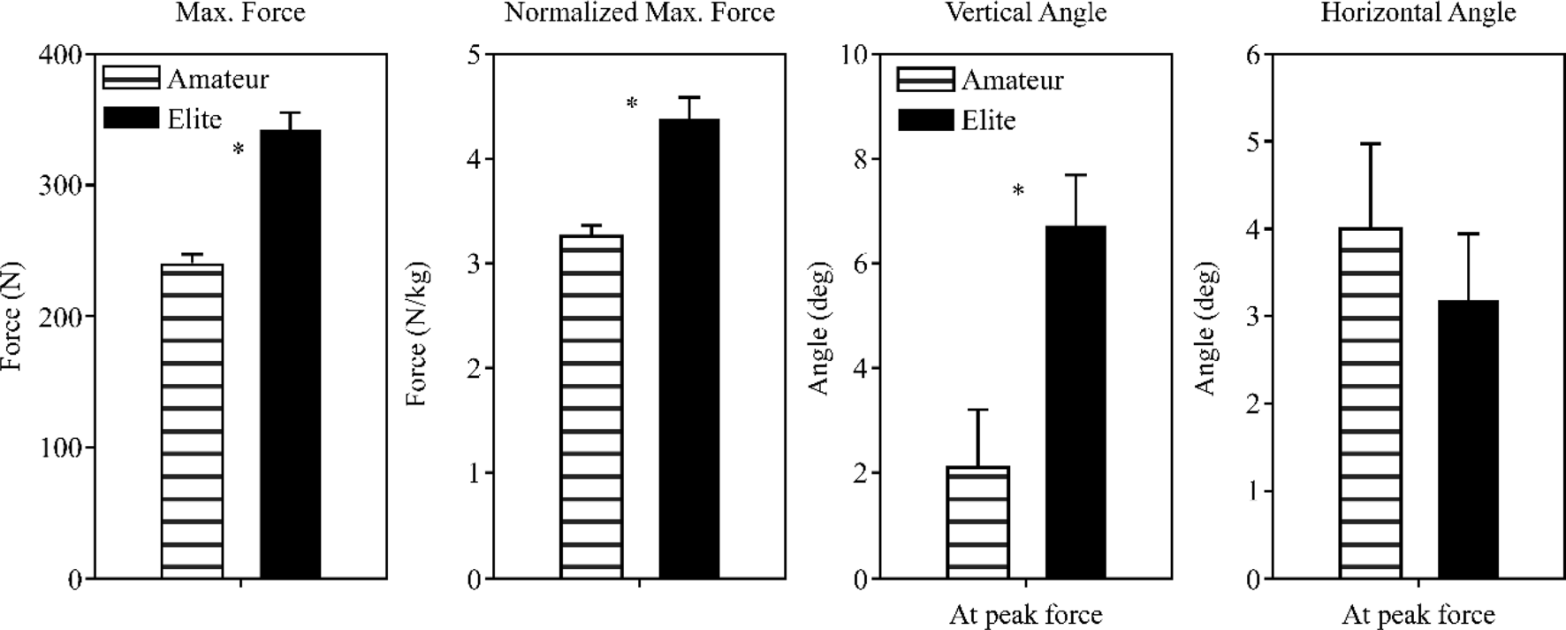
The magnitude of force during maximum force appearance (absolute value, relative value).

The absolute values of the average maximum force while performing seoi-nage were 341.1 N (SD = 66.69) and 238.3 N (SD = 36.55) for EG and NG, respectively (*t* = 6.51, *p* < 0.01). In addition, the relative values of the average maximum force were significantly different, namely 4.36 N/kg (SD = 1.02) and 3.25 N/kg (SD = 0.49) for EG and NG, respectively (*t* = 4.70, *p* < 0.01). Angles in the vertical direction (up and down) that appeared in the expression of the average maximum vector sum force of the two groups were 6.69° (SD = 4.64) and 2.07° (SD = 5.45) (EG vs. NG), thus indicating significant differences (*t* = 3.13, *p* < 0.01). In addition, angles in the horizontal direction (left, right) were 3.16° (SD = 3.79) and 3.98° (SD = 4.65) (EG vs. NG) (*t* = − 0.66, *p* = 0.512), with no significant differences between the groups (Fig. 6).

**Figure 7:**
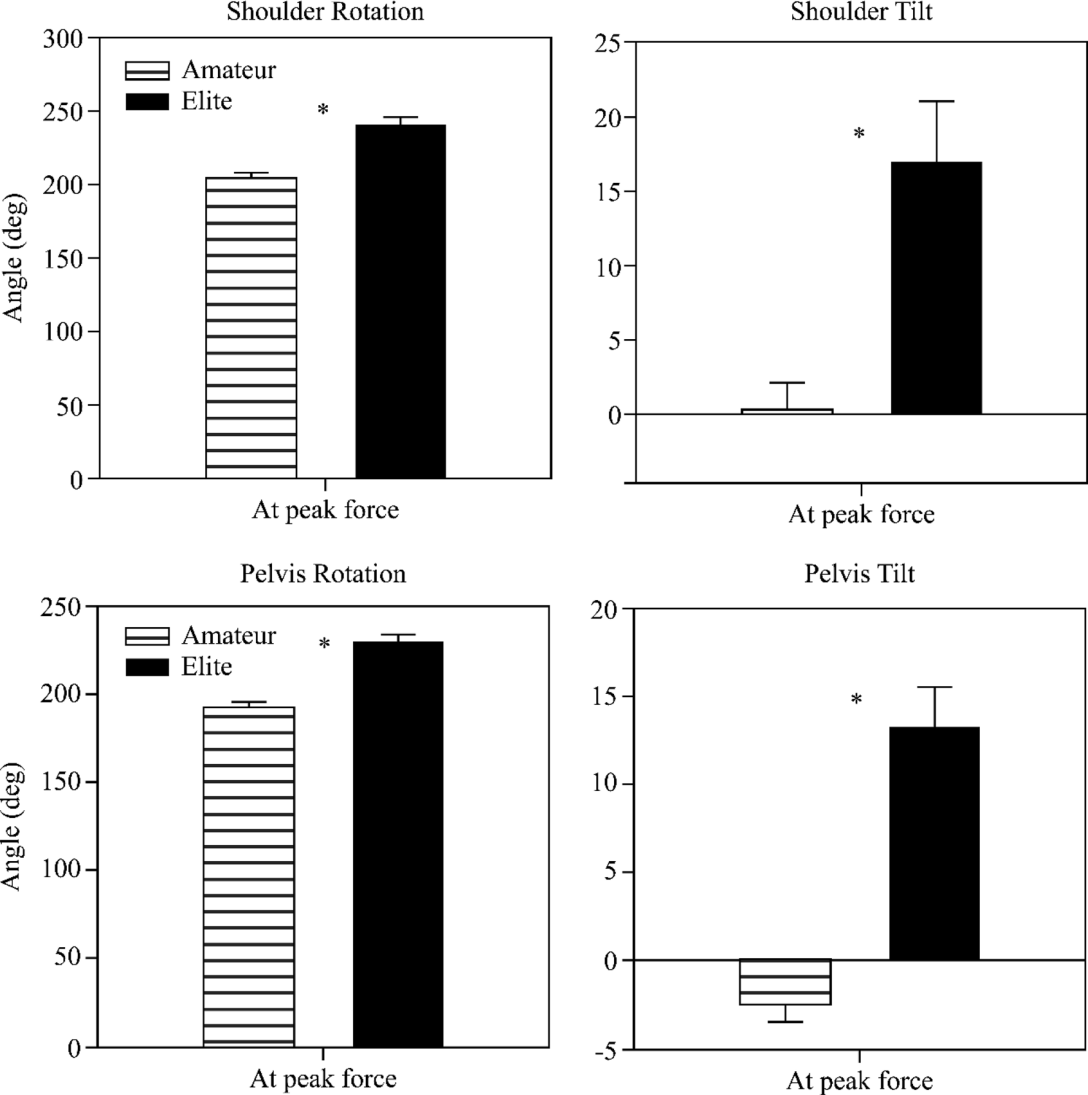
Kinematic variables at the time of maximum force appearance (shoulder, pelvis).

The horizontal rotation angles of the shoulder while performing seoi-nage were 240.8° (SD = 35.19) and 204.8° (SD = 19.89) for EG and NG, respectively, and these displayed a significant difference (*t* = 6.07, *p* < 0.01). In addition, the shoulder tilt angles were 16.92° (SD = 28.24) and 0.40° (SD = 12.94) in EG and NG, respectively (*t* = 3.63, *p* < 0.01). The horizontal rotation angles of the pelvis were 230.1° (SD = 22.62) and 191.9° (SD = 16.83) for EG and NG, respectively (*t* = 9.21, *p* < 0.01). The tilt angle of the pelvis was 13.22° (SD = 16.02) and − 2.44° (SD = 6.75) in EG and NG, respectively, indicating a significant difference between the two groups (*t* = 6.16, *p* < 0.01) (Fig. 7).

**Figure 8:**
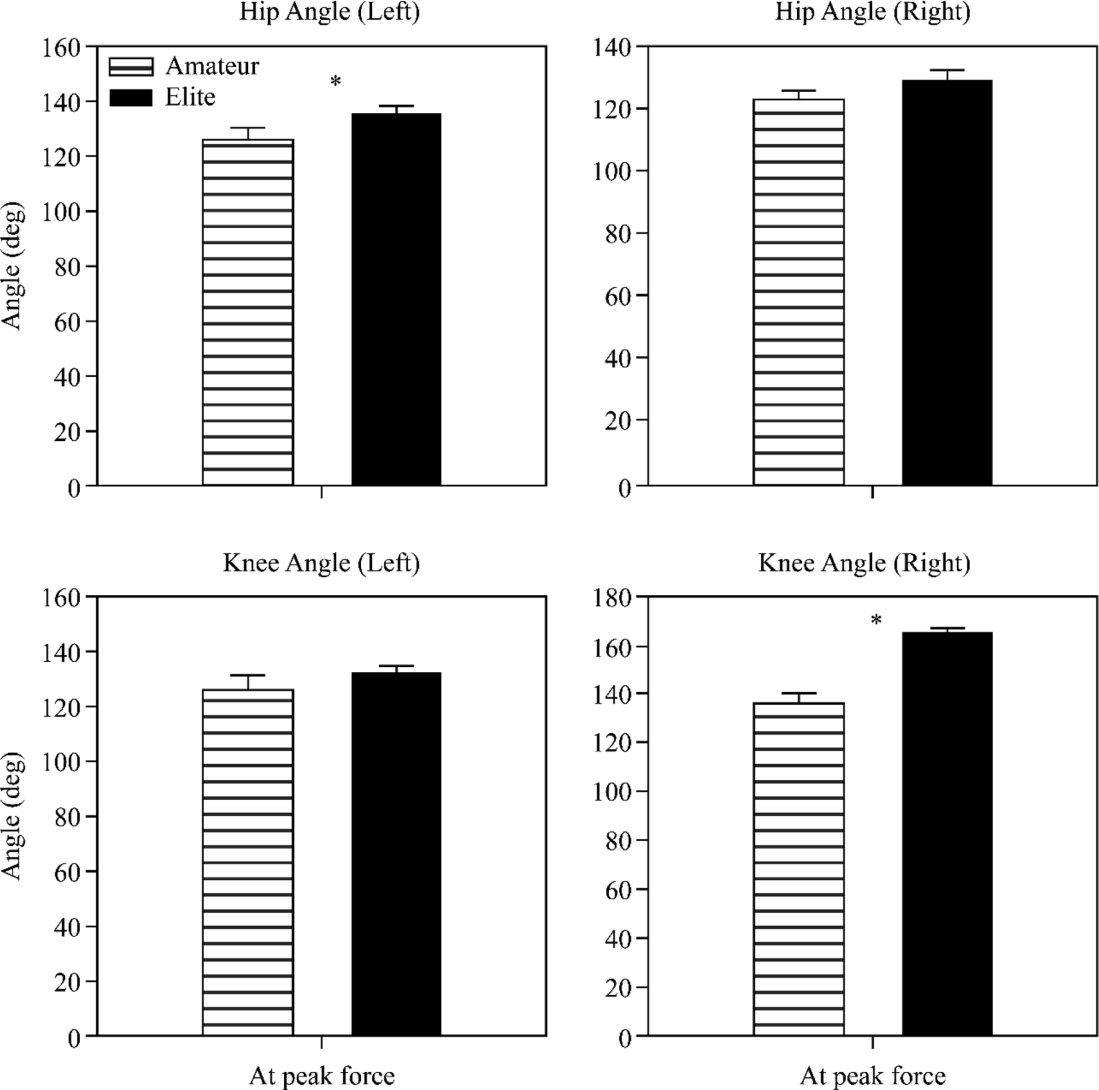
Kinematic variables at the time of maximum force appearance (hip, knee).

The left and right flexion angles of the hip joint when the maximum vector sum force appears when performing the seoi-nage, and the left hip joint angle was 135.7° (SD = 18.00) in EG and 125.8° (SD = 28.00) in NG, with a significant difference between the two groups (*t* = 2.02, *p* < 0.05); however, the right hip joint angle was 129.1° (SD = 18.91) for EG and 122.4° (SD = 25.14) for NG, and there was no significant difference between the two groups (*t* = 1.45, *p* = 0.150). The left and right flexion angles of the knee joint were analyzed. The left knee joint angle was 132.3° (SD = 15.22) in EG and 126.8° (SD = 33.93) in NG, indicating no significant difference between the two groups (*t* = 0.99, *p* = 0.325); the right knee joint angle was 165.2° (SD = 11.28) in EG and 136.0° (SD = 34.83) in NG, indicating a significant difference between the two groups (*t* = 5.36, *p* < 0.01) (Fig. 8).

## Discussion and implications

The present study investigated the kinematic characteristics of seoi-nage in elite and non-elite athletes with skill levels. We measured kinematic patterns of reactive responses with respect to seoi-nage four event movements. The main results indicated that elite athletes (EG) showed more stable balance and force control than non-elite athletes (NG). These findings indicate that the elite athletes were leading to a flexible rotation of the shoulder and pelvis. and could be drawn nearer the body with the aid of proper back shoulder and pelvic flexion.

Seoi-nage has a greater impact the better body rotation. It is known that elite athletes rotate their bodies more during seoi-nage than non-elite athletes^1,11^, according to studies by Franchini and colleagues^13^. EG had a smaller shoulder horizontal rotation angle than NG in Event 2 at the point of maximal force development. It allegedly paid more attention to pulling inside and tilting upward than shoulder turning. The shoulder and pelvic front and back tilt angles in the overall progression from Event 2 to Event 4 showed that the rear tilt was higher in EG than in NG. In addition, the front shoulder tilt was less in NG than in EG as the ability level increased. As a result, there was insufficient back-shoulder tilt for placement and unbalancing. Moreover, the front inclination of the shoulder for seoi-nage appeared little, allowing variations to be seen depending on the proficiency induced. The movement pattern of tilting at the shoulders and pelvis is supposed to demonstrate how placement and unbalance affect movement differently. In other words, proper rearward shoulder and pelvic flexion made it easier to drag the opponent toward the body. Carrying the pulled opponent forward was made possible by the front tilt of the shoulder and pelvis^8,14–17^.

The NG in the current study demonstrated force control and flexible their trunks less than did matched EG during seoi-nage. The reduced trunk and force control might be related to incoordination in the trunk and lower limb. The inertial force needed to place the opponent may be related to the range of shoulder and pelvic tilt. In all events, with the exception of Event 2 for the right hip joint, the articulation angles of the hip and knee joints were greater in EG than in NG. Due to the shoulder and pelvic posterior tilt being higher in EG than NG in Events 2 and 3, the hip joint was likewise higher in EG than NG. The left hip joint was also more stretched than the left hip joint in Event 4, which is a hang phase, and the front tilt of the shoulder and pelvis was more noticeable in the EG than in the NG. Therefore the body was kept parallel to the opponent by substantially drawing them close, despite the modest curvature of the hip joint, which caused a significant forward tilt of the shoulder and pelvis. Moreover, the heel was raised to allow the body to throw while leaning forward. The hip joint flexion following placement was not similar due to the experimental apparatus. This is due to the fact that we only measured the first phase of positioning and not its completion.These results showed that elite athletes more considerable range of tilt of the shoulder and pelvis from unbalancing to placement and the moment of inertia can also change based on the level of induced proficiency^1,18^. According to Ishii and colleagues^18^, completing seoi-nage successfully is challenging because a flexibly flexed hip joint at the beginning point of positioning did not exhibit enough angular velocity during the actual throw. Moreover, the expanded hip joint angle must be used with enough angular velocity during the early stages of placement^15,18^.

Effective seoi-nage skill requires lower limb stability and a higher-level cognitive process such as formulating the motor plan and subsequent organization of flexd step movements to environmental properties. In Event 4, EG demonstrated a more advanced knee joint angle than NG. The knee joint extension must appear to lift the opponent's center of gravity while the stance must be reduced below the knee joint^11–20^. Moreover, in all events, the right knee's flexion and extension angles were greater in EG than in NG, which is assumed to be because EG raised or pushed the opponent's center of gravity in addition to tilting and flexing the body. In Event 4, also there was a noticeable variation in the extension of EG left and right knee joints. Due to the nature of seoi-nage, the right knee extended as a result of the shoulder and pelvis rotating significantly. Some of studies, the lower right knee extension during the seoi-nage positioning phase did not demonstrate enough rotation of the shoulders and pelvis^16,21^. These findings are consistent with previous studies demonstrating that elite athletes have executed seoi-nage by sufficiently twisting the pelvis and shoulder. In addition, the left and right knee joints' flexion angles for both groups showed that the EG was more flexed or similar in Event 2 than in Event 3.

At the hip and knee joint flexion angles, EG showed a more extended pattern than NG, indicating that it looked to match the extended pattern required during the positioning phase. The maximum force vector sum in EG was 102.8 N, which was higher than in NG. This suggests that EG was more effective at pulling than NG in terms of pulling force magnitude^1,2,5,10^. In comparison to NG, EG showed higher extension in the hip and knee joint flexion angles, indicating that it behaved similarly to how extension is needed during the throwing phase. In Summary, EG had a stronger vector sum of forces than NG. Moreover, EG outperformed NG in terms of the rate of change in shoulder and pelvic tilt as well as horizontal rotation. Also, it showed hip and knee joint flexion and extension patterns that were more useful for throwing.

## Conclusion

This study identified specific kinematic characteristics of seoi-nage in reactive movement with skill levels. In conclusion, the results of this study suggest that the ability of seoi-nage influences the planning of motor execution and the implementation of motor programs within expertise. Based on the data from our study, it appears that rotation angle and motion control associated with the shoulder, pelvis, hip, and knee is an important factor that a tendency to take more trunk and lower balance control during seoi-nage. As compared with other NG, EG exhibited effective control of trunk and lower motion when seoi-nage events. The results of the present study would help to guide the development of seoi-nage strategies in elite and non-elite athletes aimed to promoting their ability to trunk and lower motion to enhance in response to skill challenges. A limitation of our study is the small sample size. Further investigations with a larger sample size are needed to validate the findings of the present study. Moreover, further study is required to determine whether such a training intervention would improve seoi-nage in non-elite athletes.

## Data Availability

The datasets used and/or analyzed during the current study are available from the corresponding author on reasonable request.

## References

[1] Ishii T, Ae M, Suzuki Y, Kobayashi Y (2018). Kinematic comparison of the seoi-nage judo technique between elite and college athletes. Sports Biomech..

[2] Hassmann M, Buchegger M, Stollberg KP (2011). Judo performance tests using a pulling force device simulating a seoi-nage throw. Ido Mov. Cult. J. Martial Arts Anthropol..

[3] Kang SK (2014). Analysis of judoist’ Game technique contents analysis in Korea national judo team (2012, London Olympic). Korean J. Phys. Eval..

[4] Kim JT, Lee DY (2011). A study on the change of COM velocity according to the type of seoi-nage in judo. J. Edu. Stud..

[5] Kim T-W, Kil S-K, Lee S-C, Jang S-H, Park S (2022). Analysis of the force characteristics of seoi-nage based on the performance height of elite male judo Athletes. Int. J. Appl. Sports Sci..

[6] Yamamoto Y, Fujii N (2019). Biomechanical study of seoi-nage in judo-influence of elbw’s pain on motion. ISBS Proc Arch..

[7] Kim EH, Yoon H (2003). A kinematic analysis of the attacking-arm-kuzushi motion as to pattern of morote-seoinage in judo. Korean J. Sport Biomech..

[8] Kim EH (2001). 3D biomechanical profiles of seoi-nage in Korean elite judoist. Korean J. Sport Biomech..

[9] Imamura RT, Hreljac A, Escamilla RF, Edwards WB (2006). A three-dimensional analysis of the center of mass for three different judo throwing techniques. J. Sports Sci. Med..

[10] Hassmann M, Buchegger M, Stollberg KP, Sever A, Sabo A (2010). Motion analysis of performance tests using a pulling force device (PFD) simulating a judo throw. Proc. Eng..

[11] Imamura R, Iteya M, Hreljac A, Escamilla R (2007). A kinematic comparison of the judo throw harai-goshi during competitive and non-competitive conditions. J. Sports Sci. Med..

[12] Ishii, T., & Ae, M. (2014). Biomechanical factors of effective seoi-nage in judo. In *ISBS-Conference Proceedings Archive*.

[13] Franchini E, Del Vecchio FB, Matsushigue KA, Artioli GG (2011). Physiological profiles of elite judo athletes. Sports Med..

[14] Barbado D, Lopez-Valenciano A, Juan-Recio C, Montero-Carretero C, van Dieën JH, Vera-Garcia FJ (2016). Trunk stability, trunk strength and sport performance level in judo. PLoS ONE.

[15] Gutiérrez-Santiago A, Prieto I, Camerino O, Anguera MT (2013). Sequences of errors in the judo throw Morote Seoi Nage and their relationship to the learning process. Proc. Inst. Mech. Eng. Part P.

[16] Kim, E. H., Park, S. J., Kang, S. Y., & Jeong, J. W. (2002). *A trunk twisting angle analysis of morote-seoinage (two arm shoulder throw) in judo* (pp. 189–200). Institute of Martial Arts, Yong-In University, *13*(1).

[17] Kort HD, Hendriks ER (1992). A comparison of selected isokinetic trunk strength parameters of elite male judo competitors and cyclists. J. Orthop. Sports Phys. Ther..

[18] Ishii, T., Ae, M., Kobayashi, Y., & Suzuki, Y. (2012). Front-turn movement in seoi-nage of elite judo athletes. In *ISBS-conference proceedings Archive 1 (1)*.

[19] Kuo, K. (2001). Comparison between knee-flexed and knee-extended styles in the major outer leg sweep. In *ISBS-conference proceedings Archive 1 (1)*.

[20] Melo SIL, Santos SGD, Piucco T, Teixeira JS (2013). Influence of judoka height when using the seoi nage technique. Revista Brasileira de Cineantropometria e Desempenho Humano.

[21] Almeida GPL, de Souza VL, Sano SS, Saccol MF, Cohen M (2012). Comparison of hip rotation range of motion in judo athletes with and without history of low back pain. Man. Ther..

